# A structured open data collection on occupant behaviour in buildings

**DOI:** 10.1038/s41597-019-0276-2

**Published:** 2019-11-26

**Authors:** Gesche Margarethe Huebner, Ardeshir Mahdavi

**Affiliations:** 1 University College London, Energy Institute, 14 Upper Woburn Place, London, WC1H 0NN UK; 2 TU Wien (Technical University of Vienna), Department of Building Physics and Building Ecology, Karlsplatz 13, 1040 Vienna, Austria

**Keywords:** Energy efficiency, Energy supply and demand, Civil engineering, Energy infrastructure

## Abstract

Climate change cannot be addressed without improving the energy efficiency of the buildings in which we live and work. The papers in this collection describe and release a series of datasets that help us understand how occupants influence and experience building energy use, both to aid future research and policy-development, and to spark wider data sharing in this important area.

Operation and construction of buildings were responsible for 36% of global final energy use and 39% of energy-related carbon dioxide (CO_2_) emissions in 2017^1^. Decarbonisation of the building stock is hence crucial in mitigating climate change. Building occupants and operators influence buildings’ energy use through their interaction with the building envelope and control systems. Systematic collection and analysis of data from buildings can support, amongst others, energy and performance contracting, smart load balancing, model-predictive building systems control, and preventive building maintenance^2^. It is also necessary for understanding, quantifying, modelling, and ultimately influencing the impact of people in buildings.

Historically, those researching occupant influences on energy use in buildings have not frequently shared data publicly. This is at odds with recent trends in other disciplines where data sharing has increased in response to the replicability crisis, i.e. the fact that attempts at replicating established findings often failed^3^ and generic principles for data sharing been introduced^4^. There are many reasons for the paucity of initiatives to make data available. One barrier is likely to be commercial sensitivity - research is often conducted together with industrial partners who might oppose data sharing. Some data cannot be shared for data protection reasons. The drive to publish in competitive academic environments might also play a role: Field data collection is often a long and expensive process and researchers might fear premature data sharing may deprive them of the rewards of their effort including scientific prestige and publication opportunities. Furthermore, data sharing requires time and effort and can expose weaknesses in data collection processes^5^.

There are many benefits of sharing data publicly beyond aiding replicability. For example, sharing data helps researchers to build on the work of others, to combine multiple data sets into larger ones allowing for an analysis with greater power, and to perform meta-analyses. For these benefits to be realized the data sets must be similar in nature and the data collection methods must be consistent. Occupant behaviour in buildings, however, is a very wide field. A set of qualitative interviews with social housing tenants can be classified as a study of occupant behaviour in buildings; as can a monitoring or modelling study of indoor air quality in offices. As such, data can be obtained via different methods, e.g. sensor-driven monitoring, interviews, focus groups, surveys, modelling, observation, and time-use diaries. Collected data may pertain to thermal comfort, occupant presence, window operation, light levels, air movement, energy use, and occupant productivity and other variables.

Discussions initially within Annex 66 of the Technology Collaboration Programme on Energy Buildings and Communities by the International Energy Agency (https://annex66.org/) and then continued under Annex 79 (http://annex79.iea-ebc.org/) highlighted the challenges of comparing different studies due to the variety of methods and data ontologies used. The need for structured approaches to the collection, storage, sharing, and analyses of monitored data relevant to building occupants became a key focus of work in these Annexes resulting in books and articles on the subject. Motivated by the absence of publicly available and generally agreed-upon building monitoring data ontologies, Mahdavi *et al*.^2,6^ proposed a general ontology for the representation and incorporation of multiple layers of monitored building data in pertinent computational applications. The proposed ontology entails six basic data categories, namely *i)* inhabitants, *ii)* indoor environmental conditions; *iii)* external environmental conditions; *iv)* control systems and devices, *v)* equipment, and *vi)* energy flows. Each of these basic categories have multiple subcategories (see Fig. 1).

**Fig. 1:**
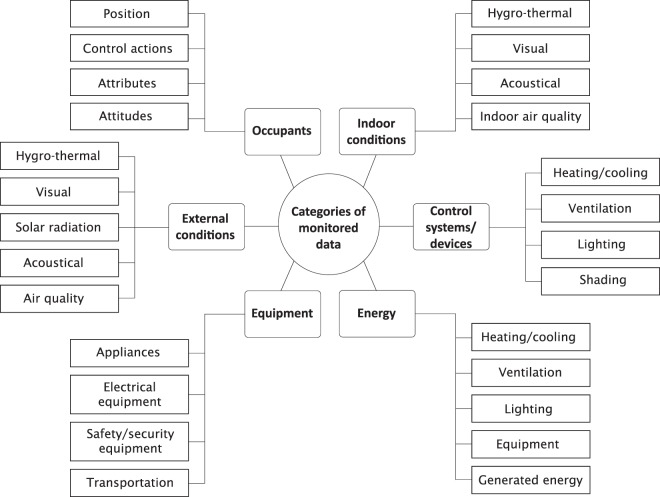
Categories and sub-categories of building monitoring data. This figure displays categories and sub-categories of building monitoring data (based on^6^) as considered in the development of the building monitoring ontology^2^.

In this ontology, the ‘Occupants’ category has four subcategories: (1) the position of occupants in the space (which hence indicates the presence of occupants), (2) control actions such as opening windows, (3) people’s attributes, e.g., their activity and clothing levels, and (4) people’s attitudes, e.g. their – often self-reported – thermal comfort assessment. Indoor conditions category captures spatial characteristics in hygro-thermal, visual, acoustical, and indoor air quality domains. External conditions can have a direct impact on internal conditions (e.g. through solar radiation) and an indirect one through occupants’ actions (e.g. closing windows to avoid traffic noise exposure). Control systems and devices category captures the state of systems for heating, cooling, ventilation, lighting, and shading as indicated, for instance, in terms of thermostat set-points and blind positions. The equipment category refers to any technical entities meant to support occupants’ activities (such as communication devices, computers, electric appliances) but are not meant to control indoor environmental conditions. Energy category refers to magnitudes of energy provided to (or generated in) the buildings. Monitored data resolution can be defined: (1) in spatial terms (e.g. rooms, floors, and whole buildings), (2) across multiple systems (e.g. heating, lighting, and equipment), and (3) in temporal terms (e.g. hourly, daily, monthly).

All submissions to the special collection on ‘Occupant Behaviour in Buildings’ were requested to map the collected data onto the ontology proposed by Mahdavi *et al*., to encourage streamlining of reporting and to allow future users to see synergies between various data descriptors. The ontology proposed by Mahdavi *et al*. also suggests a specific structure on how variables from those six categories should be specified in terms of their values, associated sources, and possible actors. However, data descriptors in the present collection were not instructed to strictly conform to this.

Langevin present data from a one-year study of 24 U.S. office focusing on indoor conditions, occupants’ attitudes, and control systems. In daily surveys, occupants reported on their thermal comfort, preference and behaviour. Sensors recorded local thermal environmental and control states. In total, 2503 survey responses alongside tens of thousands of concurrent behaviour and environment measurements were generated^7^.

Mahdavi *et al*. present data monitored over a period of one year in an office area in a university building in Vienna. The collected data includes occupants’ presence and operation of lights and windows. Moreover, to provide a suitable interpretative context, monitored indoor environmental conditions (temperature, humidity) as well as plug loads are included in the data set, together with outdoor environmental parameters (i.e., temperature, humidity, global irradiance, wind speed, and wind direction)^8^.

Paige *et al*. present a data set from a longitudinal affordable housing study collected in six affordable housing units in the USA where the performance of these units did not meet the design target (net-zero energy standard). The authors provide energy data at a 1 Hz sampling rate for four circuits: main, hot water heater, dryer, and HVAC. They also report variables from the occupants category (i.e., attitudes, actions, and position), external conditions (i.e. weather), and control systems (e.g. heating type)^9^.

Schwee *et al*. focused on indoor conditions and occupant presence, collecting room-level data on occupant counts, airflow, CO2, relative humidity, illuminance, and temperature data covering three rooms, one lecture room, and two study zones. The dataset consists of 47 full days^10^.

Schweiker *et al*. present monitoring data collected over a period of four years from a naturally ventilated low-energy office building located in Frankfurt, Germany. The data set includes indoor and outdoor environmental conditions, energy-related information, as well as occupants’ presence and behaviour^11^.

Schweiker *et al*. questioned the underlying assumptions of the measurement tool routinely used to assess thermal comfort, i.e. within the category of occupants’ attitudes. The authors collected data in 30 countries on the assumption of equidistance of the ASHRAE thermal comfort scale by asking participants to state the perceived distance between the verbal anchors of the survey questions. In addition to testing the assumption of equidistance, this dataset allows to analyse the influence of different contexts (e.g. language, climate, or season) and characteristics of individuals (e.g., males/females)^12^.

We believe the present contribution represents the first open collection of data on occupant behaviour in buildings structured following the same data ontology. Needless to say, we welcome and encourage future contributions to this body of work. Ultimately, we hope to establish a common process for data reporting that may become more widely used over time and help the research community to reap the benefits of data sharing, including facilitation of meta-analyses, generation of large samples, and aiding replicability.
